# Patient information leaflets for placebo-controlled surgical trials: a review of current practice and recommendations for developers

**DOI:** 10.1186/s13063-024-08166-x

**Published:** 2024-05-22

**Authors:** S. Cousins, M. Huttman, N. Blencowe, C. Tsang, D. Elliott, J. Blazeby, D. J. Beard, M. K. Campbell, K. Gillies

**Affiliations:** 1 National Institute for Health Research Bristol Biomedical Research Centre Surgical Innovation Theme, Bristol Centre for Surgical Research, Bristol Medical School, University of Bristol, Bristol, UK; 2 Royal College of Surgeons Surgical Trials Centre Bristol, University of Bristol, Bristol, UK; 3 Royal College of Surgeons Surgical Trials Centre Oxford, University of Oxford, Oxford, UK; 4 Royal College of Surgeons Surgical Trials Centre Aberdeen, Health Services Research Unit, University of Aberdeen, Aberdeen, UK; 5 Health Services Research Unit, University of Aberdeen, Aberdeen, UK

## Abstract

**Introduction:**

Informed consent for participation in an RCT is an important ethical and legal requirement. In placebo surgical trials, further issues are raised, and to date, this has not been explored. Patient information leaflets (PILs) are a core component of the informed consent process. This study aimed to investigate the key content of PILs for recently completed placebo-controlled trials of invasive procedures, including surgery, to highlight areas of good practice, identify gaps in information provision for trials of this type and provide recommendations for practice.

**Methods:**

PILs were sought from trials included in a recent systematic review of placebo-controlled trials of invasive procedures, including surgery. Trial characteristics and data on surgical and placebo interventions under evaluation were extracted. Directed content analysis was applied, informed by published regulatory and good practice guidance on PIL content and existing research on placebo-controlled surgical trials. Results were analysed using descriptive statistics and presented as a narrative summary.

**Results:**

Of the 62 eligible RCTs, authors of 59 trials were contactable and 14 PILs were received for analysis. At least 50% of all PILs included content on general trial design. Explanations of how the placebo differs or is similar to the surgical intervention (i.e. fidelity) were reported in 6 (43%) of the included PILs. Over half (57%) of the PILs included information on the potential therapeutic benefits of the surgical intervention. One (7%) included information on potential indirect therapeutic benefits from invasive components of the placebo. Five (36%) presented the known risks of the placebo intervention, whilst 8 (57%) presented information on the known risks of the surgical intervention. A range of terms was used across the PILs to describe the placebo component, including ‘control’, ‘mock’ and ‘sham’.

**Conclusion:**

Developers of PILs for placebo-controlled surgical trials should carefully consider the use of language (e.g. sham, mock), be explicit about how the placebo differs (or is similar) to the surgical intervention and provide balanced presentations of potential benefits and risks of the surgical intervention separately from the placebo. Further research is required to determine optimal approaches to design and deliver this information for these trials.

**Supplementary Information:**

The online version contains supplementary material available at 10.1186/s13063-024-08166-x.

## Introduction

There are ethical and legal requirements for potential participants to provide their informed consent to be involved in a randomised controlled trial (RCT). The informed consent requirements for RCTs include both verbal and written information (informed consent forms or documents and/or patient information leaflets (PILs) depending on the jurisdiction of the host country). The informed consent information for RCTs as a minimum, explains: the purpose and aim of the trial, research procedures, anticipated risks and benefits, end-of-trial provisions, source of funding, potential conflicts of interest and researchers’ institutional affiliation [1]. Potential participants must also be made aware of their right to refuse and the ability to withdraw their consent at any time [1]. Sometimes the informed consent process requires particular additions. One area is placebo trials. Regulatory guidance recommends explaining what a placebo is, that these are used solely for research purposes, and providing details of the potential risks/discomforts linked to randomisation to placebo [2, 3]. They also recommend informing patients that placebos may be indistinguishable from the treatment intervention [4]. However, this overarching guidance is limited and does not contain explicit details of the information that should be provided to patients about invasive placebo interventions, such as those used in surgical trials.

Previous reviews examining PILs for placebo-controlled trials showed that PILs lacked information regarding potential non-therapeutic benefits and risks arising from receiving a placebo intervention were not fully explained, possibly reflecting the limited guidance in this area [5, 6]. These reviews focused on trials of pharmaceutical placebos alone, which are fundamentally different from surgical trials. First, placebos used in surgical trials are often invasive requiring access to the body (via incision, natural orifice or percutaneous puncture) and may involve anaesthesia [7]. As such, the potential risks associated with taking a ‘sugar pill’ as a pharmaceutical placebo may be minimal, whereas the potential physiological insult associated with placebos in surgical trials has additional considerations. As such, there are associated ethical issues regarding potential risk and acceptability to patients, which have obvious implications on information provision to potential participants [8].

General guidance for placebo-controlled surgical trials has suggested enhanced information to participants and consideration of expanded consent needs [9–11]. However, the details of how this should be operationalised have not been specified. The recent ASPIRE guidance has gone further and outlined some key content for PILs in trials with an invasive placebo comparator [12]. This includes recommendations to provide the following: a full description of the invasive placebo procedure; a statement that whilst there may be some benefit of undergoing the placebo procedure, it is not designed to confer benefit; recognition that the use of the placebo is for research only; and information on potential risks for both the placebo comparator and surgical procedure. The ASPIRE guidance also recommends that language that may promote therapeutic misconception should be avoided, including the use of terms such as ‘sham’ or ‘fake’ surgery [12].

It is currently unknown what information about invasive placebo comparators (and the surgical intervention) is included in PILs to support patients’ decision-making about participation in trials of this type. As such, this study aimed to investigate the content of PILs for completed placebo-controlled trials to highlight areas of ‘good’ practice but also identify gaps in information provision for trials of this type and as such develop recommendations based on current practice.

## Methods

### Sampling

A previously conducted systematic review had identified 96 placebo-controlled randomised trials of invasive procedures (covering endoscopy, minimal access, percutaneous and open surgery) [7]. From this sample, the main RCTs published from 2002 (to coincide with the introduction of the European Clinical Trials Directive 2001/20/EC [2]) to 2019 (pre ASPIRE guidance to allow future comparison of the impact of the guidance on PILs) were included if they recruited adults 18 years or older with the capacity to make a decision about their own participation. Pilot/feasibility studies, cluster RCTs, RCTs conducted in an emergency setting, and those recruiting participants under 18 years of age were excluded.

### Data collection

The trial teams of all eligible RCTs were systematically approached and asked to provide a copy of the written information provided to patients in the trial. Initially, an email was sent to the corresponding author from a research fellow (SC). This was followed up with a reminder email after 1 week and a telephone call after 2 weeks. If the corresponding author’s email was unavailable or no longer valid, a web search for the most recent institutional affiliation was undertaken to find an alternative email. Failing this, web searches were undertaken to find contact details for an alternative author contact (the first or senior author, whichever had not already been approached) and the process was repeated.

### Eligibility

Written patient information leaflets for individual patients, which in some instances included consent forms if a separate PIL was not available or the content information was presented in the consent form rather than the PIL, were eligible for inclusion, i.e. written information that supports the initial decision to participate in the RCT, or not. Documents not wholly related to patient information provision were not included. Recruitment or publicity posters, documents providing instructions to patients already recruited to the trial (e.g. post-op instructions), and those where patients were not the primary intended audience were excluded.

### Data extraction and analysis

Key trial characteristics were extracted, including first author, year of publication, geographical region, number of centres, number of patients randomised, surgical specialty and a brief description of the surgical intervention under evaluation. The degree of fidelity (high, medium or low) of the placebo comparator to the surgical intervention under evaluation was also assessed. As per the ASPIRE guidance ‘Varying levels of fidelity are possible and include low fidelity, in which there is little similarity to the complete surgical intervention (e.g., skin incisions only, thus resembling what surgeons would have traditionally described as a sham treatment), and maximum fidelity, in which treatment occurs with a complete set of surgical attributes (i.e., the surgical procedure under evaluation). In between these extremes, a high fidelity placebo might have identical surgical content and attributes to the complete surgical procedure but does not have the presumed active or essential component. A medium fidelity placebo might have fewer surgical components and less resemblance to the complete surgical procedure. A no surgery control has no attributes of the index procedure.’ [7]. Judgements were informed by deconstructing both interventions into their constituent components to examine to what extent placebo and surgical interventions were matched in components delivered [13]. Judgements were made by two reviewers independently and any disagreements were resolved by discussion with the research team. Data were summarised using descriptive statistics, where appropriate.

### Content analysis of PILs

Information relating to both the invasive placebo and active intervention included in each PIL was analysed using a directed content analysis approach. Directed content analysis is guided by a structured approach that uses existing theory (or research) to identify key concepts as initial categories and sub-categories within a framework [14]. Initial categories were informed by constructs identified from guidance, expert knowledge and a review of a sub-set of included PILs. A Microsoft Excel spreadsheet listing the framework categories/sub-categories was used to facilitate content analysis and to map verbatim text from the PILs to categories. Four researchers (SC, DE, NB, KG) applied the preliminary framework to three included PILs. Allocation of text was compared between reviewers and category/sub-category definitions, additions and subtractions refined through discussion. The resulting final framework was then applied to all PILs, with 20% coded by an independent second reviewer (MH). Where more than one eligible PIL was received for an RCT (e.g. a ‘brief’ PIL with an accompanying longer document), content was analysed on a per RCT, rather than per document, basis.

Both the quantitative (frequency of information items within PILs) and the qualitative (text content mapped to each category/sub-category) data are presented as a narrative summary, highlighting aspects of good practice (based on existing guidance) and areas for improvement. The data is also presented visually as a ‘heat-map’ in Table 2 where green indicates areas of high content coverage, red indicates low content coverage and grey indicates where content is absent.

## Results

### Included PILs

**Fig. 1:**
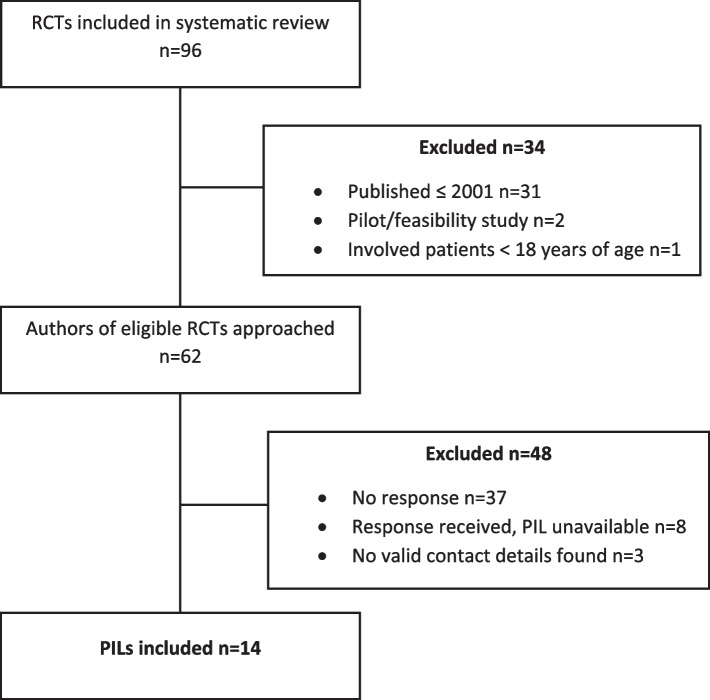
Sampling, approach and responses from eligible RCTs from Cousins et al. [7]

Figure 1 shows included RCTs and responses from authors approached. Sixty-two RCTs were eligible for inclusion from the original sample of 96 [7]. We were able to email the authors of 59 trials and 14 responded (response rate 24%) with PILs from trials published between 2005 and 2018.

### Characteristics of RCTs of included PILs

Of the included trials, the majority were trials with sites only in Europe (*n* = 8, 57%) and they involved various surgical specialties (Table 1). In one case, PILs were provided from two RCTs testing the same intervention within the ear, nose and throat. Sample sizes across the 14 RCTs ranged from 22 to 313 (median = 77) and where the number of centres was reported this ranged from 1 to 36. Most (*n* = 10) included a high-fidelity placebo comparator, i.e. one which closely resembles the surgical procedure under evaluation except that the key component has been removed.

**Table 1:** Characteristics of included PILs

PIL ID	Publication year	Region	No. centres	No. patients randomised	Surgical specialty	Fidelity of placebo	Invasive intervention (verbatim term/s used to describe group)	Placebo comparator (verbatim term/s used to describe group)
1	2005	Europe	NR	26	Oral and maxillofacial	High	Radiofrequency (RF) surgery of the soft palate for snoring (‘one of the treatment groups’)	RF device inserted but not activated (‘dummy’; ‘one of the treatment groups’)
2	2008	USA	3	100	Oral and maxillofacial	High	Palatal implants for obstructive sleep apnea (‘treatment’)	Identical implementation device used without an implant (‘placebo’)
3	2009	Australia	4	78	Orthopaedics and trauma	Medium	Percutaneous injection of polymethylmethacrylate for osteoporotic vertebral fractures (‘real’)	Injection of anaesthetic but no cement (‘placebo’; ‘mock’)
4	2009	USA	NR	76	Neurosurgery	Low	Surgical deactivation of migraine headache trigger site (‘actual surgery’)	Exposure of muscles and nerves without changing their integrity (‘placebo’; ‘control’)
5	2010	Multi-region	30	288	Cardiothoracic surgery	High	Bronchial thermoplasty (‘treatment’)	Bronchoscopy and insertion of thermoplasty device but with no energy applied (‘control’; ‘sham’)
6	2012	Europe	1	22	Oral and maxillofacial	High	Palatal implants for obstructive sleep apnea	Identical implementation device used without an implant (‘mock’)
7	2014	USA	7	214	Gastrointestinal surgery	Medium	Sphincterotomy for pain after cholecystectomy (‘active’)	Endoscopy and endoscopic retrograde cholangiopancreatography (‘control’)
8	2014	Europe	1	35	Oral and maxillofacial	High	Radiofrequency surgery of the soft palate (‘fairly new method’; ‘treatment with […] radiowaves’)	Radiofrequency wand inserted but no energy applied (‘treatment […] without radio waves’)
9	2014	USA	36	277	Cardiothoracic surgery	High	Intra-bronchial valve (‘study procedures’)	Catheter inserted with no device (‘study procedures’; ‘no study valves group’)
10	2015	Europe	NR	50	Cardiothoracic surgery	High	Endobronchial valves (‘active’)	Bronchoscopy with no valve placement (‘control’)
11	2015	Europe	5	44	Gastrointestinal surgery	Medium	Transoral incisionless fundoplication using EsophyX device in gastro-oesophageal reflux disease (‘experimental’; ‘investigational’)	Endoscopy (‘sham’; ‘control’)
12	2017	Europe	32	313	Orthopaedics and trauma	High	Arthroscopic subacromial decompression (‘type of surgery’)	Arthroscopy (‘type of surgery’)
13	2017	Europe	1	72	Ear, nose and throat	High	Inferior turbinate surgery with radiofrequency ablation (‘surgical treatment’)	Device not turned on (‘placebo’)
14	2018	Europe	5	200	Interventional cardiology	High	Percutaneous coronary intervention (‘treatment’)	Coronary angiogram (‘medical treatment alone’)

### Frequency of information items within PILs

Overall, most of the PILs in the sample included general items on trial design with at least 50% of all PILs covering content such as the purpose of including a placebo; blinding (and timing of unblinding) of the trial team and participants; and whether those receiving placebo will be offered the intervention at end of study (Table 2). The most frequently reported items within the PILs were within the intervention description section, with both a description of components of the placebo and a description of the intervention being reported in all PILs analysed. Yet, explanations of how the placebo differs or is similar to the surgical intervention (i.e. fidelity) were only reported in 6 (43%) of the included PILs.

**Table 2:**
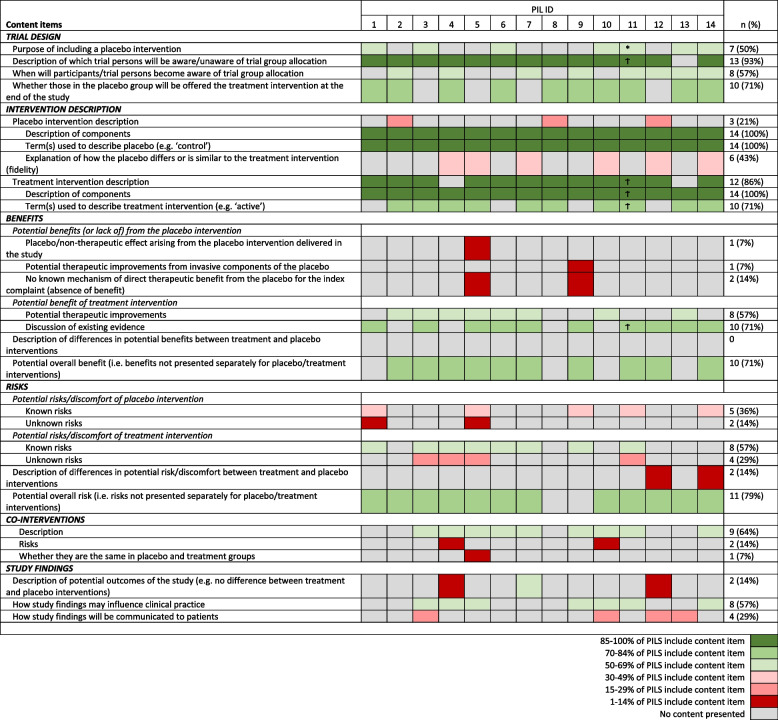
Analysis of content items across PILs

^1^Content analysis included both Pre-op Information and consent form

*Data present in pre-op info only

ϮData present in consent pre-op info and consent form

When considering the presence of information on the risks and benefits of the placebo comparator and the surgical intervention, the majority (57%) of the PILs included information on the potential therapeutic benefits of the surgical intervention. One (7%) of the PILs included information on potential indirect therapeutic improvements from invasive components of the placebo, with a further PIL including information on the potential non-therapeutic effects of the placebo intervention. With regard to risk information, again only a small number (*n* = 5, 36%) presented the known risks of the placebo intervention, whilst 57% (*n* = 8) presented information on the known risk of the surgical intervention. Only two PILS included descriptions of the differences in potential risks between surgical and placebo interventions. Most of the included PILs did not separate the potential benefits or risks associated with the placebo or the surgical interventions individually.

The following section of the findings considers the specific content items identified in the frequency analysis of information content of the placebo surgical trials PILs.

### Purpose of including a placebo intervention

Of the 14 included PILs, seven (50%) included information on the purpose of including a placebo intervention in the trial. One PIL stated that conflicting results from previous studies were largely due to participants being unblinded and over/underestimating effects on outcome. Other PILs stated that the purpose was to ‘be absolutely certain of the value of’ the surgery, or to ‘allows us to make a proper comparison of the effect without being biased’ so as ‘to scientifically and objectively evaluate a treatment method of this kind’. One PIL gave the following justification:

This particular study design is the only possible way to ultimately provide evidence whether the treatment is effective and to establish that the observed outcomes are not down to a so-called “placebo effect”.

### Term(s) used to describe placebo and surgical interventions

All 14 PILs included terms to describe the placebo intervention. A range of different words were used as descriptors, which included (and some PILs used more than one term to describe): *control* (*n* = 3); *placebo* (*n* = 3); *mock [surgery]* (*n* = 2); *sham* (*n* = 2); ‘*treatment without [intervention]*’ (*n* = 2); *dummy treatment* (*n* = 1); *medical treatment alone* (*n* = 1); *placebo surgery* (*n* = 1); and *type of surgery* (*n* = 1). Terms used to describe the treatment intervention with the PILs focussed on *treatment* (*n* = 3), *active* (*n* = 2), *actual surgery* (*n* = 1), *experimental (investigational) device or procedures* (*n* = 1), *new* (*n* = 1), *real surgery* (*n* = 1) and *surgical treatment* (*n* = 1);

### Explanation of how the placebo differs or is similar to the surgical intervention (fidelity)

Content on placebo fidelity (i.e. how the placebo differs or is similar to the surgical intervention) was included in a number of PILs. These PILs discussed this in terms of how the procedure was similar to the surgical intervention, e.g. ‘Apart from the shaving away of a small amount of bone that sits above the tendons, the operation is very similar to the shoulder arthroscopy (above)*.*’ (high fidelity placebo), and ‘if you are assigned to the control group, you will be treated in the same way as patients in the actual surgery group before and after surgery. The muscles or the nerves, however, will not be removed.’ (low fidelity placebo). However, within some PILs the content described the difference between the surgical intervention and placebo as a dichotomy, e.g. ‘the other group (called a “control” group) will NOT receive a sphincterotomy’ (medium fidelity placebo).

### Inclusion of information on benefits and risks of placebo and surgical intervention

Only one PIL included content in relation to potential placebo/non-therapeutic effects from the placebo intervention, stating: ‘Sometimes, people feel better due to a “placebo effect”—that is, they expect to feel better because something may have been done to help their condition’. The study that included information on the potential therapeutic improvements from invasive components of the placebo (in this case a diagnostic bronchoscopy) stated that it could lead to a diagnosis of (lung) cancer or detect ‘potential airways abnormalities, polyps, or foreign objects’. Information about known risks related to the placebo intervention focussed on the risks associated with anaesthesia or the intervention or the combination, e.g. ‘Rarely, there are allergic reactions to the local anaesthesia.’; ‘those patients who are allocated to the medication only group may require a third procedure in which they have coronary stenting. This will be an additional risk and will be the same as that of routine coronary angioplasty (1 in 100 risk of major bleeding, death, heart attack or stroke following coronary angioplasty).’; and ‘The risks associated with bronchoscopy are related to sedation and the procedure itself’*.*

## Discussion

This study has systematically analysed the content of PILs for placebo-controlled trials of invasive procedures including surgery in relation to information relevant to informed consent. The analysis focussed on determining how key content related to the placebo controls was described and presented in comparison to the surgical interventions. Whilst most PILs in the sample described the purpose of including a placebo, and, whether those receiving a placebo will be offered the intervention at the end of the study, there was an imbalance across other items. In particular, content on risks and benefits of placebo comparators was routinely not described yet are a content item described in some informed consent guidance, e.g. FDA requirement [3].

Existing studies that have analysed PILs for placebo-controlled trials, albeit largely pharmaceutical trials, have also found similar results to this study. A review by Hernández and colleagues found that in the majority of the patient information sheets they analysed, the placebo was clarified although no explanation about its risks or related adverse reactions was given [6]. No information was found in any of the included study documents about the placebo effect (i.e. the nocebo effect) and 6% (23/359) lacked any information about placebos [6]. Another study which conducted an analysis of 45 PILs from placebo-controlled drug trials also found that placebos were described less often than ‘treatments’, and potential benefits and adverse effects were also reported more infrequently for the placebo comparators [5]. Our findings are similar to those published from non-surgical studies confirming the generalisability, but also allow further reflection on the unique findings from this work on placebo-controlled surgical trials, such as issues around the description of fidelity, which would not be as relevant for placebo-controlled drug trials.

The explanation of how the placebo differs or is similar to the treatment intervention, i.e. fidelity, was not well described in our sample of PILs. Yet our analysis determined that 10 of the PILs were from trials for which the placebo intervention could be considered as having high fidelity to the surgical intervention. It is, however, important to recognise that items describing fidelity may not be applicable to all placebo surgical trials and will be dependent on the nature of the placebo [12]. The similarity of the placebo to the surgical intervention under evaluation (i.e. placebo fidelity) should be key in considering what to include in the information shared with potential participants. This could help to address concerns over the therapeutic misconception in trials of this type [15]. For example, when considering a placebo with no fidelity (i.e. a procedure that does not resemble the treatment procedure in any way except a skin incision), it would be necessary to inform potential participants that allocation to a placebo may not result in personal therapeutic benefit. Conversely, a high-fidelity placebo (i.e. one which closely resembles the surgical procedure under evaluation except that the key component has been removed) may confer some potential therapeutic benefit to the participant, even if not designed as such, and so needs to be framed accordingly.

Again considering that the fidelity of the placebo to the surgical intervention was considered ‘high’ across 10 of the PILs in the sample, it would be expected that the known risks and benefits of the placebo would be presented given its high fidelity of the surgical intervention. However, only 5 (36%) of PILs presented the known risks of the placebo intervention. Other research has evidenced that potential trial participants rate information on the potential disadvantages and risks of taking part highly when considering trial participation [16]. Presentation of risk information in PILs for clinical trials more generally has not been done as well as it could. An analysis of PILs regarding the presence of information on presenting probabilities (which could be for risks or benefits) demonstrated that none of the PILs in the sample analysed provided this information—an aspect which has been deemed important for high-quality decision-making [17, 18]. However, this is not surprising given there is no high-quality evidence or agreement on how best to present risk information in PILs for potential trial participants [19]. Further research to understand how PILS describe the magnitude of effects or the influence of framing effects could be helpful. There is work on addressing this issue through the development of principles for sharing information about the potential benefits and harms of trial interventions with potential trial participants [20]. It will be important to consider the findings of this work for its applicability to the context of placebo-controlled surgical trials. There is evidence, however, that involving potential trial participants during PIL development can help with clarity of information, ensure it is neither discriminatory nor stigmatising and consider culturally appropriate and sensitive methods of presentation and delivery [21, 22]. This further supports the need to involve patients as partners in the research process and critically in processes supporting informed consent for trials.

### Strengths and limitations

Whilst the included sample of 14 PILs was relatively small and likely influenced by responder bias, the included sample still demonstrates variability and at the level of overall characteristics is largely similar to the trials included in the systematic review. However, it must be acknowledged that the PIL sampling is limited by the review, with no new search to update the included studies beyond 2017, and therefore is the potential for selection bias to have influenced findings. It is also important to note that in trials where PILs were not available, this was often linked to issues of data retention (e.g. not held for longer than 10 years, staff moving on and others not knowing where/how to access, sponsor companies merging and data not being available). These data retention issues need to be addressed to ensure the rigour of future methodological projects based on the data held within the original trials. A key strength is the inclusion of trials from across a range of surgical specialties and over a 13-year time horizon. However, the restriction of PILs up to 2019 may have resulted in the omission of some more recent studies that demonstrated closer alignment to ‘good’ practice. Yet we plan to investigate this temporal trend through a future analysis post-2020 to examine the impact of ASPIRE publication. Given many of the PILs included in this analysis were from trials designed more than 10 years ago (i.e. even the most recent trial included from 2018 likely initially developed the PIL in 2013), it will also be important to consider how the evidence supporting decision-making for trial participation has progressed thinking in the past decade and how this might influence PIL development going forwards. A further strength was the high level of methodological rigour in the analysis given all PILs were double-coded.

### Key recommendations

Based on the findings from our study and the broader literature, we have developed general recommendations for consideration when writing PILs for placebo-controlled surgical trials.

- Critically consider the use of language, specifically how certain terms (e.g. mock/sham) may cause misunderstanding or concern. Also, consider if there is an adequate understanding of the term placebo especially when it is a high-fidelity comparator—it may not mean doing nothing.
- Information should be provided about how the placebo differs or is similar to the treatment intervention. What aspects of the intervention will not be done in the placebo arm, what will be done differently and what remains the same?
- The presentation of potential benefits and risks of placebo intervention should be more balanced to be comparable with the risks and benefits presented for the surgical intervention. This will be influenced by the level of fidelity of the intervention and comparator where low fidelity interventions may be expected to have very different risk/benefit profiles but higher fidelity interventions possibly being more similar. Ensuring any potential risks and benefits are presented using existing methods of good risk communication, e.g. presentation of probabilities, could also be followed to promote high-quality decision-making.
- Ensure there is early involvement of patients and/or public partners in the development of content for the PIL. Consider what the core content items are (which could be guided by those defined in Table 2) and ask patients to consider what information should be presented and how to enable informed choices about participation to be made.

Whilst a number of key bodies (e.g. American Medical Association and the Royal College of Surgeons England) have called for the need for ‘enhanced’ consent and/or patient information for placebo-controlled surgical trials, all trials must ensure that all relevant information is included to ensure informed consent, and thus it may be more important to first address the potential omissions with regard to fidelity, and risk/benefit information. Applying a level of ‘enhanced’ process assumes there is a greater risk, which may not be the case, and may have potential unintended consequences for recruitment trials of this type.

## Conclusions

This study has analysed the information content of PILs used during informed consent for placebo-controlled surgical trials. The findings add to the existing research on best practices for the information content of PILs for clinical trials more broadly but add new insights with regard to key content for trials of this type and reveal important areas for improvement. Developers of PILs should carefully consider the use of language (e.g. sham, mock), be explicit about how the placebo differs (or is similar to) the surgical intervention and provide balanced presentations of potential benefits and risks of the surgical intervention separately from the placebo. Further research is required to determine optimal approaches to design and deliver this information for these trials and gather patient experience on the process of consent for placebo-controlled surgical trials.

### Supplementary Information

Supplementary Material 1: Supplementary Table 1. Comparison of characteristics of trials included in systematic review (reference 7) and PIL analysis.

## Data Availability

The data that support the findings of this study are available from the authors of the trials included in Cousins et al. [7].
