# Supplementary material for: Patient information leaflets for placebo-controlled surgical trials: a review of current practice and recommendations for developers
Source: Trials. 2024 May 22;25:339. doi: 10.1186/s13063-024-08166-x (PMC11110406; doi:10.1186/s13063-024-08166-x)
Supplement: Supplementary file 1 — Supplementary Material 1: Supplementary Table 1. Comparison of characteristics of trials included in systematic review (reference 7) and PIL analysis. [file 13063_2024_8166_MOESM1_ESM.docx]

**Supplementary Table 1. Comparison of characteristics of trials included in systematic review (reference 7) and PIL analysis**

| **Characteristic** | | **Number of RCTs in review^7^ (%)** | **Number of PILs (%)** |
| --- | --- | --- | --- |
| Year of publication | ≤2000 | 29 (30) | 0 |
|  | 2001–2010 | 39 (41) | 5 (36) |
|  | 2011–2017 | 28 (29) | 9 (64) |
| Region | Europe | 40 (42) | 8 (57) |
|  | United States | 37 (36) | 4 (29) |
|  | Australia | 4 (4) | 1 (7) |
|  | Canada | 3 (3) | 0 |
|  | Asia | 2 (3) | 0 |
|  | Not specified | 1 (1) | 0 |
|  | Multiregion | 9 (9) | 1 (7) |
| Clinical area | Gastrointestinal | 40 (42) | 2 (14) |
|  | Orthopedics & trauma | 15 (16) | 2 (14) |
|  | Oral and maxillofacial | 10 (10) | 4 (29) |
|  | Cardiothoracic | 7 (7) | 3 (21) |
|  | Ear, nose, and throat | 6 (6) | 1 (7) |
|  | Interventional cardiology | 5 (5) | 1 (7) |
|  | Neurosurgery | 5 (5) | 1 (7) |
|  | Other[^a^](https://www.sciencedirect.com/science/article/pii/S0895435619307449?via%3Dihub#tbl1fna) | 8 (8) | 0 |
| Number of centres | 1 | 31 (32) | 3 (21) |
|  | 2–5 | 17 (18) | 4 (29) |
|  | 6–10 | 7 (7) | 1 (7) |
|  | >10 | 16 (17) | 3 (21) |
|  | Not reported | 25 (26) | 3 (21) |
| Number of patients randomised | 1–100 | 65 (68) | 9 (64) |
|  | 101–200 | 16 (17) | 1 (7) |
|  | >200 | 14 (15) | 4 (29) |
| Treatment intervention | Endoscopic | 44 (46) | 5 (36) |
|  | Minimal access | 21 (22) | 2 (14) |
|  | Percutaneous | 20 (21) | 6 (43) |
|  | Open surgery | 11 (11) | 1 (7) |

^7^ Cousins, S., N. S. Blencowe, C. Tsang, A. Lorenc, K. Chalmers, A. J. Carr, M. K. Campbell, J. A. Cook, D. J. Beard and J. M. Blazeby (2020). "Reporting of key methodological issues in placebo-controlled trials of surgery needs improvement: a systematic review." J Clin Epidemiol 119: 109-116.
